# Characterization of individuals with chronic pain: phenotyping approaches used in MAPP

**DOI:** 10.1186/1744-8069-10-S1-O19

**Published:** 2014-12-15

**Authors:** David A Williams

**Affiliations:** 1 Department of Anesthesiology, University of Michigan, Ann Arbor, MI 48105, USA

## 

Pain is a complex perception affecting one’s state of consciousness, functional status, and quality of life. Clinically, pain intensity is often the only facet of pain that is assessed; but two large research networks (i.e., the Orofacial Pain: Prospective Evaluation and Risk Assessment (OPPERA) [1] and the Multi-Disciplinary Approach to the Study of Chronic Pelvic Pain (MAPP) [2]) have characterized pain in accordance with broader pain concepts. MAPP was designed to characterize individuals with urologic chronic pelvic pain syndromes (UCPPS) which consisted of diagnoses such as interstitial cystitis, bladder pain syndrome, chronic prostatitis, and chronic pelvic pain syndrome. Characterization of the sample of n=1039 individuals occurred at multiple sites and at multiple levels of analysis including: biomarkers, self-report questionnaires, quantitative sensory testing (QST), functional neurobiological studies, and structured and resting state neuroimaging studies. A comprehensive assessment occurred at baseline followed longitudinally by biweekly or bimonthly internet questionnaires and more extensive in-clinic visits at 24 and 48 weeks following baseline [3]. The self-report methods covered both urologic-specific and non-urological domains relevant to chronic pain which are consistent with the bio-psycho-social model of pain. Urological domains included urological diagnostics, symptoms and impact, sexual functioning, self-esteem, and social relationships. Non-urological-specific self-assessment included clinical pain, functional status, mood, co-morbid conditions, personality, attitudes/ beliefs, and early life trauma. Biological specimens linked to clinical data included cheek swabs and plasma, as well as urine for exploration of infectious etiology. Also linked to the clinical data was quantitative sensory testing (QST) which helped to characterize individuals with respect to pain threshold, and neuroimaging studies providing structural, functional and network connectivity data corresponding to central pain processing and modulation. Ultimately, the goal of such extensive phenotyping will be to identify subgroups of individuals with UCPPS who have distinct underlying pathophysiology to which more optimal treatment approaches can be aligned thus offering the potential for improved disease management and improved patient care.

## Disclosures

Consultant for Health Focus Inc.

## References

[B1] Maixner W Diatchenko L Dubner R Orofacial pain prospective evaluation and risk assessment study – the OPPERA study J Pain 2011 12 T4 11 10.1016/j.jpain.2011.08.002 22074751 PMC3233836

[B2] Clemens J The MAPP research network: A novel study of urologic chronic pelvic pain syndromes BMC Urology 2014 14 57 10.1186/1471-2490-14-57 25085007 PMC4134515

[B3] Landis JR The MAPP research network: Design, patient characterization, and operations BMC Urology 2014 14 58 10.1186/1471-2490-14-58 25085119 PMC4126395

